# In situ laser manipulation of root tissues in transparent soil

**DOI:** 10.1007/s11104-021-05133-2

**Published:** 2021-09-12

**Authors:** Sisi Ge, Lionel X. Dupuy, Michael P. MacDonald

**Affiliations:** 1 School of Science and Engineering, University of Dundee, Nethergate, Dundee, DD1 4HN UK; 2 Neiker, Department of Conservation of Natural Resources, Berreaga 1, 48.160, Derio, Spain; 3 The James Hutton Institute, Invergowrie, Dundee, DD2 5DA UK; 4 IKERBASQUE, Basque Foundation for Science, Plaza Euskadi 5, 48009 Bilbao, Spain

**Keywords:** Transparent soil, Root, *Lactuca sativa*, Laser dissection, Imaging

## Abstract

**Aims:**

Laser micromanipulation such as dissection or optical trapping enables remote physical modification of the activity of tissues, cells and organelles. To date, applications of laser manipulation to plant roots grown in soil have been limited. Here, we show laser manipulation can be applied in situ when plant roots are grown in transparent soil.

**Methods:**

We have developed a Q-switched laser manipulation and imaging instrument to perform controlled dissection of roots and to study light-induced root growth responses. We performed a detailed characterisation of the properties of the cutting beams through the soil, studying dissection and optical ablation. Furthermore, we also studied the use of low light doses to control the root elongation rate of lettuce seedlings (*Lactuca sativa*) in air, agar, gel and transparent soil.

**Results:**

We show that whilst soil inhomogeneities affect the thickness and circularity of the beam, those distortions are not inherently limiting. The ability to induce changes in root elongation or complete dissection of microscopic regions of the root is robust to substrate heterogeneity and microscopy set up and is maintained following the limited distortions induced by the transparent soil environment.

**Conclusions:**

Our findings show that controlled in situ laser dissection of root tissues is possible with a simple and low-cost optical set-up. We also show that, in the absence of dissection, a reduced laser light power density can provide reversible control of root growth, achieving a precise “point and shoot” method for root manipulation.

## Introduction

The growth of plant roots is highly sensitive to small changes in the surrounding soil environment. Even when plants are grown under controlled environments, root developmental parameters remain highly variable (Adu et al. 2014). The soil itself is heterogeneous, and many of its properties exhibit variations that are known to influence root growth. Humidity gradients affect tropisms, pore sizes and compaction change the morphology and anatomy of the root, whilst nutrient distribution modulates developmental parameters such as lateral root initiation and elongation rate (Martins et al. 2020; Tracy et al. 2012; Unger and Kaspar 1994). Currently, our ability to study and understand such responses remains limited, and new techniques are needed to manipulate plant roots and monitor their responses in situ.

Non-destructive imaging techniques have greatly enhanced our ability to observe physical interactions between plant roots and their surrounding environments. The instruments available to perform neutron radiography (Rudolph-Mohr et al. 2014), MRI and X-ray microtomography (Gregory et al. 2003; van Dusschoten et al. 2016) can now track water movements and resolve soil structure and root anatomical traits at unprecedented resolution, but direct manipulations of the root itself are more limited. It is difficult, for example, to measure the internal mechanical stresses building up on a growing root in response to soil mechanical resistance (Bengough et al. 2011) or to control the number of bacteria attaching to epidermis cells (Romano et al. 2020).

Remote micromanipulation using physical fields such as light fields, magnetic or acoustic fields provides high levels of control on environmental cues affecting the activity of multicellular organisms, cells or organelles. Laser beams can be used, for example, for dissection and suppressing signals from neighbouring cells (Schou et al. 2002), to place pathogens in contact with host cells (Tam et al. 2010), or even manipulate chloroplasts inside a living plant cell (Li et al. 2015). Acoustic approaches have been used to control the plant transpiration rate (Gomez et al. 2017) or to measure the mechanical properties of cell walls (Gadalla et al. 2014). Magnetic fields have been used to manipulate organelles inside living cells by controlling embedded magnetic beads within the cells (De Vries et al. 2005). Application of such techniques to root tissues grown in soil is more challenging because the heterogeneity of the substrate affects the geometry and efficacy of the fields.

**Fig. 1:**
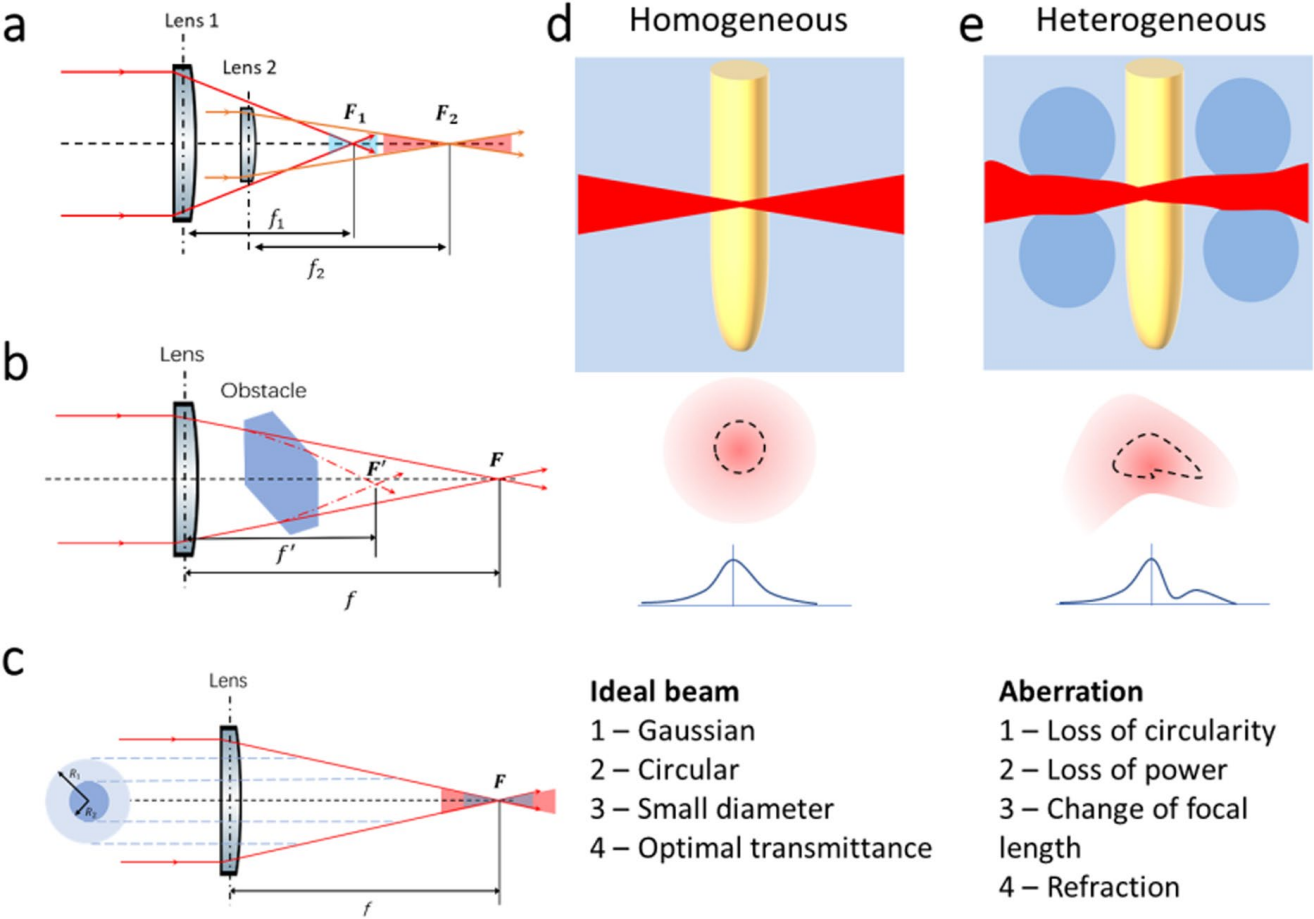
Factors affecting the precision of laser dissection. **a**) Lenses can affect the properties of the beam through their Numerical Aperture (NA). At the focal point of a lens, the thickness *d* of the beam cutting is controlled by Abbe’s diffraction formula $d=\frac{\lambda }{2 NA}$ . Therefore, a lens with a large numerical aperture (Lens 1, blue) produces higher energy density and larger cut area near the focal point, but the Rayleigh range (axial range for which the beam is well focussed) and hence the depth of cut is reduced with comparison to the small numerical aperture (Lens 2, red). **b**) Changes in the refractive index of the medium affect the optical path and focal distance of the lenses, but also the amount of light reflected and scattered by particles. **c**) The increase of laser beam output power increases the volume where the cutting energy density is reached, and the overall volume of the cut obtained is larger. Therefore, if a higher laser power is used, cutting expands further away from the laser focal point. d-e) The medium itself affects the properties of the cut. A homogeneous substrate preserves the Gaussian properties of the beam (**d**), maintaining constant cutting size and shape. Heterogeneity induces aberrations, brought largely by refraction, with direct implications on precision of the dissection (**e**)

The recent development of synthetic polymer substrates which mimic soil conditions (transparent soil, TS) has opened new avenues of research on live microscopy of roots and associated microorganisms. The system was used successfully to culture a range of plant species, bacteria (Downie et al. 2012) and free-living nematodes (O’Callaghan et al. 2018). Furthermore, it has also been demonstrated that it is compatible with modern microscopy techniques (Sharma et al. 2020; Yang et al. 2013). Fluoropolymers used in the fabrication of transparent soils have excellent mechanical, optical and thermal stability, which indicates the possibility of using higher power pulsed and continuous-wave lasers for in situ dissection and other types of optical manipulation, provided that any aberrations induced by soil heterogeneity don’t excessively reduce laser beam quality (Fig. 1).

In this study, we investigated whether optical manipulations can be performed on living roots within the transparent soil itself. We characterised the influence of soil heterogeneity on the quality of the laser beam, and quantified the nature of distortions introduced by the soil and determined optimal parameters for application to plant roots. Finally, the application of optical manipulation is demonstrated in two study cases, namely in situ dissection and growth inhibition.

## Methods

### Laser manipulation instrument

**Fig. 2:**
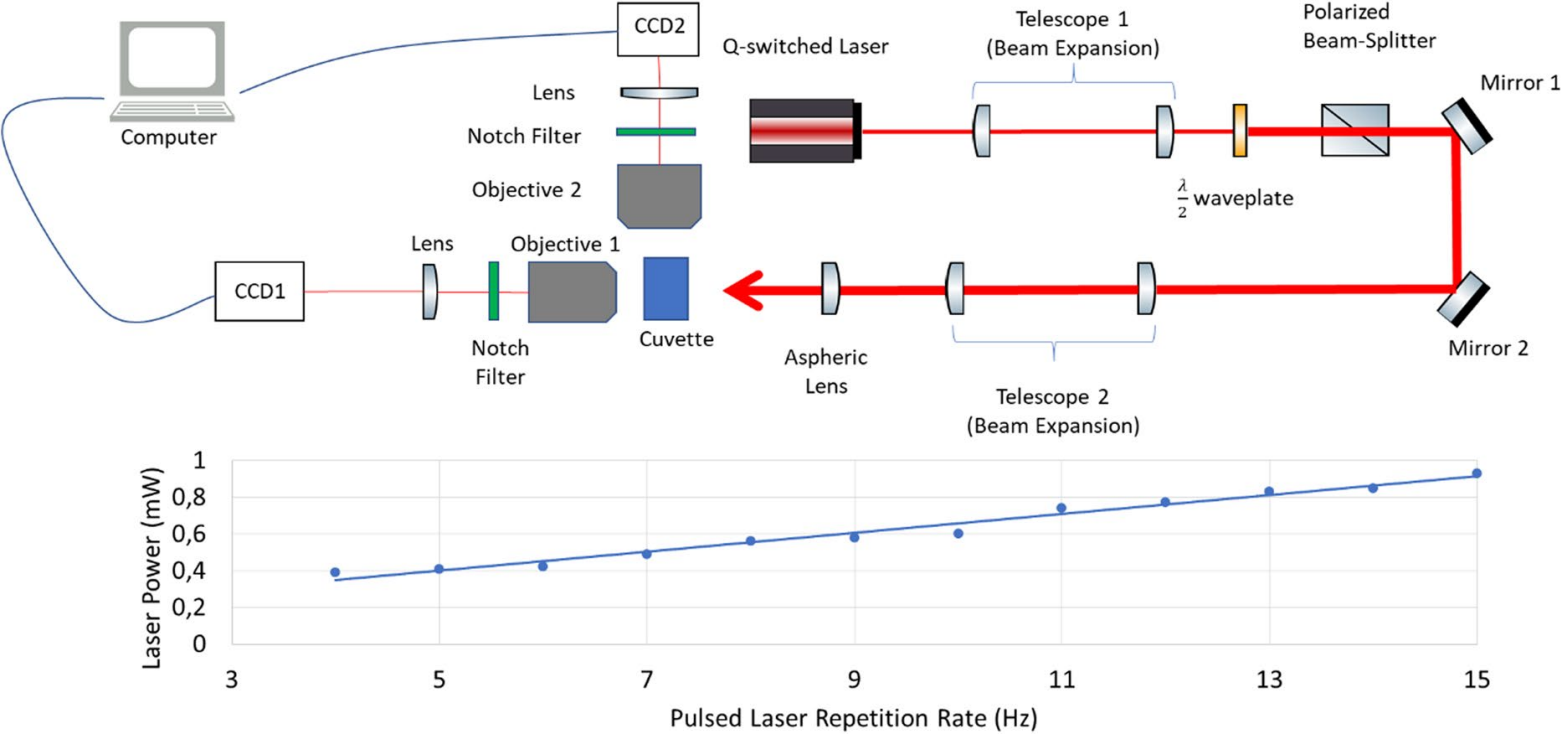
Laser dissection instrument. A Q-switched laser is first passed through a telescope (telescope 1) for beam expansion. A $\frac{\lambda }{2}$ waveplate is used to control the power output of the laser beam. A second telescope is subsequently used for further laser beam expansion when a larger back aperture aspherical lens is used; and for beam steering by making a mirror conjugate to the back of the focusing lens or objective. Near the focal point of the beam, the optical density is maximal and dissection occurs. Two imaging arms are used in the set-up. The first imaging arm (objective 1) captures images along the beam axis and is used for analysis of the beam profile. The second imaging arm (Objective 2) captures images perpendicular to the beam axis and is used to measure the depth of the dissection. The power output of the laser beam is controlled either by the repetition rate or through the combined effect of the $\frac{\lambda }{2}$ waveplate and the polarising beam splitter. A Köhler illumination system produces the illumination field (Madrid-Wolff and Forero-Shelton 2019)

The laser dissection instrument (Fig. 2) utilises a low-cost frequency-doubled Nd:YAG Q-switched laser operating at 532 nm wavelength (Continuum minilite II, Photonic Solutions, UK) with a pulse repetition rate between 1 Hz and 15 Hz and peak power of 5.0 × 10^6^ W to 8.3 × 10^6^ W. Plano-convex lenses (LA1131-A, LA1509-A, Thorlabs, UK) were used to collimate and expand the laser beam. Various aspheric lenses [(C220TMD-C, f = 11.00 mm, NA = 0.25); (C240TME-C, f = 8.0 mm, NA = 0.5); (C280TMD-C, f = 18.40 mm, NA = 0.15) Thorlabs, UK] were used to focus the beam for cutting. We used a half waveplate (WPH05M-532, Thorlabs, UK) combined with a polarising beamsplitter (CCM1-PBS251/M, Thorlabs, UK), to regulate the power output of the laser beam. A linear stage with 13 mm travel range (M-562-XYZ, Newport, UK) was used to move samples and control cutting depth and speed. Cuvettes containing seedlings were anchored on the linear stage using several mounting bases (BA1/M and BA1S/M, Thorlabs, UK). The two imaging arms consisted of an objective (TU Plan ELWD 20X, Nikon; M Plan Apo SL 20X, Mitutoyo, UK), a notch filter (NF533–17, CWL = 533 nm, FWHM = 17 nm, Thorlabs, UK), which is used to block the laser scattering light from reaching the camera (Allied Vision, Guppy Firewire F-146). A side-view imaging arm was used for observation of root dissections. We assembled a Köhler illumination system for brightfield imaging using a fibre illuminator (OSL1-EC, Thorlabs, UK). The brightness and area of illumination are adjusted by the diaphragm (SM2D25D, Thorlabs, UK) at a constant field of view.

### Measurements of the effect of the substrate and laser output power on the properties of the beam

We analysed the geometrical properties of laser beams from the shape and area cut on a test sample using a method similar to the traditional “burn paper” technique (Boyd et al. 2008). Test samples consisted of thick black vinyl sheets of the thickness (100 ± 3 μm, National Sign Company, UK) attached to a microscope slide (631–0113, VWR, UK). Once the laser reaches the vinyl, a cut occurs with the properties of the laser beam directly measurable through the cross-section of the cut, with cutting only possible at locations with sufficient optical power density. The laser was operated with a pulse repetition rate of between 5 Hz and 12 Hz (0.30 mW - 0.77 mW) on eight different locations on the vinyl. The average laser power corresponding to the pulse repetition rate can be found in Fig. 2.

To study the effect of the growth substrate on the properties of the cutting beam, a chamber was assembled with a microscope slide and a coverslip (631–0125, VWR, UK) separated by 16 layers of black vinyl sheets. The 1.6 mm space created by the vinyl sheets was filled with transparent soil or agar gel and clamped on the translation stage 2 mm ahead of the focal point of the laser cutting beam. For each substrate (air, gel and transparent soil), cuts were made in 10 different locations on the vinyl sheet, and the cuts were analysed for surface area, diameter and circularity as described in the data analysis section.

Various aspheric lenses were fitted to study how, if at all, beam properties affect the cutting width and depth in relation to laser power. For each configuration of laser output and focusing lens, dissections were carried out on eight different locations on the vinyl. All cuts were subsequently analysed for surface area and circularity as described in the data analysis section.

### Plant growth

Lettuce seeds (*L. sativa*) were surface sterilised in 10% bleach for 20 min before multiple washes in distilled water were applied. Seedlings were germinated overnight in agar. Following germination, transparent soil was placed in square glass cuvettes (CV10Q3500F, Thorlabs, UK), and two seedlings were gently sowed at the surface of each cuvette. Seedlings were transferred to transparent soil when radicles were between 1 mm to 3 mm long. The top of the cuvette was sealed with parafilm tape (PM996, Cole-Parmer, UK). When root growth was studied in agar, seeds were germinated directly in glass cuvettes. Plants were grown at room temperature (approximately 15 °C) under natural light. Monitoring of root growth started approximately 10 h after the transfer. Time-lapse image data of root growth was recorded for 36 h, with one image acquired every two hours. After 18 h of growth, samples were placed in the dissection instrument to expose the root to the laser beam.

### Transparent soil

Transparent soil was prepared as previously described in Downie et al. (2012). Nafion® pellets (Ion Power Inc., USA) were fractured in a cryogenic mill (SPEX SamplePrep 6770) to sizes of 250 μm to 1250 μm. Particles were converted to anionic form by washing in 6 M KOH, 35% 5 M DMSO at 80 °C and subsequently in 3 M nitric acid at room temperature. Murashige and Skoog Basal Medium (Sigma M5519) at 4.4 g L^−1^ was used to titrate Nafion® particles and subsequently autoclaved for 20 min at 121 °C.

### Study case 1: Laser dissection in situ

Laser dissection has proven essential to understand how cells and tissues exchange signals and coordinate their activity during development (Nakazono et al. 2003), or study the mechanical stress created by growing organs (Dumais and Steele 2000). Such processes are essential to understand how roots adapt to constraints from the soil environment, but performing such analysis non-destructively is difficult. Hence, we first used the optical manipulation instrument to test whether the dissection of root tissues can be made efficiently, live and in situ.

To perform the dissection of roots and quantify the cutting depth, a total of 24 plants were sown in 24 cuvettes. 12 samples were first used to characterise dissection depth in air following removal from the gel. A further 12 samples were sown to study dissection in agar and transparent soil. Out of the 6 samples, 3 were discarded because the root grew in the centre of the cuvette, and the substrate attenuated the power of the laser beam. The samples were placed vertically on the stage of the dissection instrument. The height of the sample was adjusted using the brightfield image. The sample stage was translated horizontally at a speed of (74 ± 7) μm s^−1^. The movement of the translation stage caused the root to move across the path of the laser beam and induced a transverse cut into the root tissue. Cuts were performed at powers ranging from 0.2 mW to 0.8 mW. The depth of the cut was then measured from image data by calibrating the recorded video against a graticule.

### Study case 2: Growth inhibition using low light doses

The elongation rate of a root is a primary factor affecting the microbial colonisation of the rhizoplane (Hodge et al. 2009). The root elongation rate can be modified through external application of growth hormones or other chemical compounds, but such approaches are indiscriminate. To test whether root elongation rate can be reduced by local application of laser light, we studied root responses to exposure to low light doses.

In this experiment, 32 root samples were sowed in 16 cuvettes. 8 cuvettes contained agar and the remaining 8 cuvettes contained transparent soil. 10 h after germination, roots that exhibited delayed growth or grew in the centre of the cuvette were discarded. For each treatment, 8 roots showing a strong elongation rate were subsequently used for the experiment. To reduce the intensity of the laser beam, and to increase the area of the root exposed to the laser beam, root samples were placed at approximately 100 μm from the focus of an aspheric lens (C280TMD-C). The laser beam was applied near the root tip in the zone of the rapid increase in diameter (cell division zone). The exposure consisted of a 15 s horizontal scan of the beam across the root tip at a translation speed of approximately 20 μm s^−1^. The laser beam was used at a 15 Hz pulse repetition rate (0.93 mW average power). Video recording resumed following exposure to the laser.

### Data analysis

The holes cut into the black vinyl were analysed using ImageJ (Abràmoff et al. 2004). A fixed threshold was first applied to identify pixels corresponding to a cut. A region of interest was selected on the image before using an edge tracing algorithm to extract information on the size and shape of the cut. The position of the centre of mass of the cut (*x*_*i*_, *y*_*i*_), was extracted from the image data. Since samples were moved horizontally, spatial variations induced by the heterogeneity of the transparent soil was assessed by measuring the variance of the vertical coordinate of the centre of mass,

1 $$v=\frac{1}{n}\sum {\left({y}_i-\overline{y}\right)}^2$$

Time-lapse images of lettuce roots before and after exposure to light were used to quantify the change in root elongation rate resulting from exposure to the laser beam. Samples were removed from the dissecting instrument, and images were obtained using a different camera sensor (C930e, Logitech, USA). Image data was calibrated for size, and the root length was recorded manually using segmented lines. *e*_*i*_ the elongation rate at time step *i* was calculated as

2 $${e}_i\left(\mathrm{mm}\ {\mathrm{h}}^{-1}\right)=\frac{L_{i+1}-{L}_i}{dt},$$

where *L*_*i*_ (mm) is the length of the root at the time *t*_*i*_, and *dt* is the time increment (here 2 h). The mean elongation rate $\overline{e}$ was then calculated from data before and after exposure to the laser,

3 $$\overline{e}=\frac{1}{n}{\sum}_i{e}_i,$$

The ratio between elongation rate before exposure ($\overline{e_{before}}$) and after exposure ($\overline{e_{after}}$) was calculated as

4 $$r=\frac{\overline{e_{after}}}{\overline{e_{before}}}.$$

## Results

### Effect of power and numerical aperture on the properties of the dissection

**Fig. 3:**
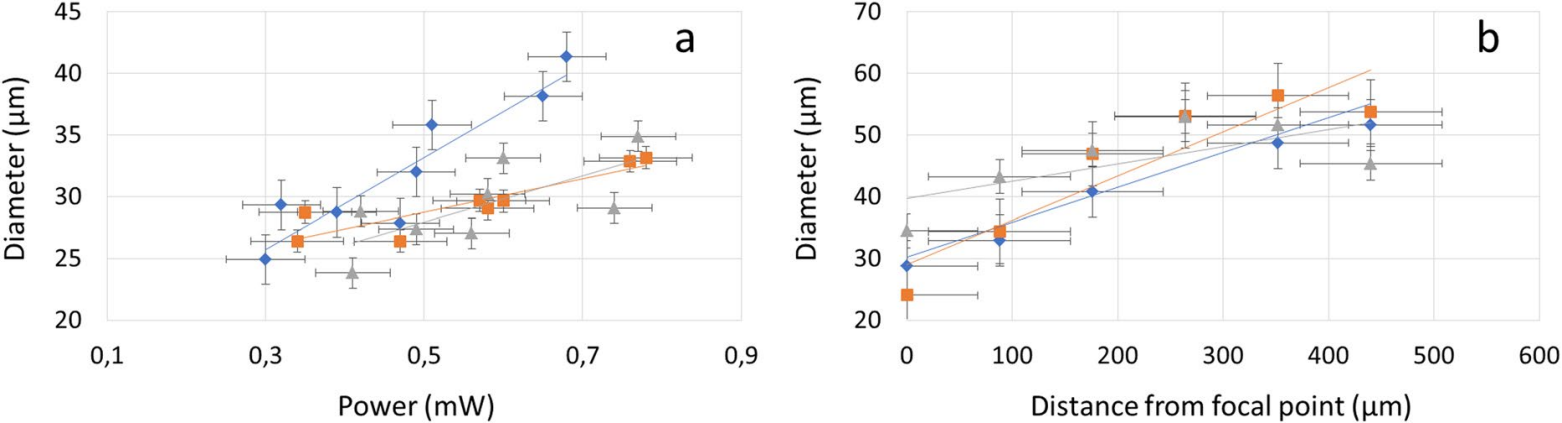
Effect of the power output of the laser beam and distance from the focal point on the precision of dissection. (**a**) The power output increases the diameter of the cut, and the focusing lenses can affect this response. (**b**) The diameter of the cut obtained with 0.5 mW of power output increased with the distance from the focal point, and the type of focusing lens did not affect this relationship

To investigate the robustness of laser manipulation to the type of microscopy set-up and if necessary to optimise the operating parameters, the power output of the laser beam, as well as the lens used for focusing the beam on the sample, were varied. We used three different aspherical lenses (C220TMD-C; C240TME-C; C280TMD-C) and tested them with various configurations of power output of the laser beam and distance from the focal point. The average diameters of the cut on the vinyl sheet at 0.3 mW to 0.78 mW of average power were (32 ± 5) μm for the lens C220TMD-C; (29 ± 2) μm for the lens C240TME-C; (29 ± 3) μm for the lens C280TMD-C (Fig. 3a).

The power of the laser had a strong influence on the area of material cut at the focal point. Within the range of settings tested, the diameter of the hole cut by the laser beam was linearly correlated with the power output of the laser beam, and the distance from the focal point had a strong effect on the area of the vinyl cut by the laser beam (Fig. 3b).

By translating the vinyl sample along the beam axis to various distances from the focal point of the laser beam, we observed that cutting was maintained within a 600 μm range from the focal point, with the increase of the distance from the focal distance resulting in an increase in the diameter of the hole cut into vinyl. For example, at 0.58 mW of average output power, the hole cut into the vinyl became distorted when the distance from the focal point reached approximately 352 μm, indicating the most efficient cutting region of the laser beam. The focusing lens had a limited effect on the cutting depth at a given power level, which indicates the technique can be performed under various microscopy environments and the properties of the cut can be controlled solely using the power outputs of the beam.

Overall, these results indicate the potential of the dissection instrument for use in diverse applications, including for dissection through heterogeneous substrates. Cutting remained robust to changes such as the distance from the focal point, and across a range of beam focusing lenses of various focal lengths and numerical aperture.

### Effect of substrate heterogeneity on laser dissection properties

**Fig. 4:**
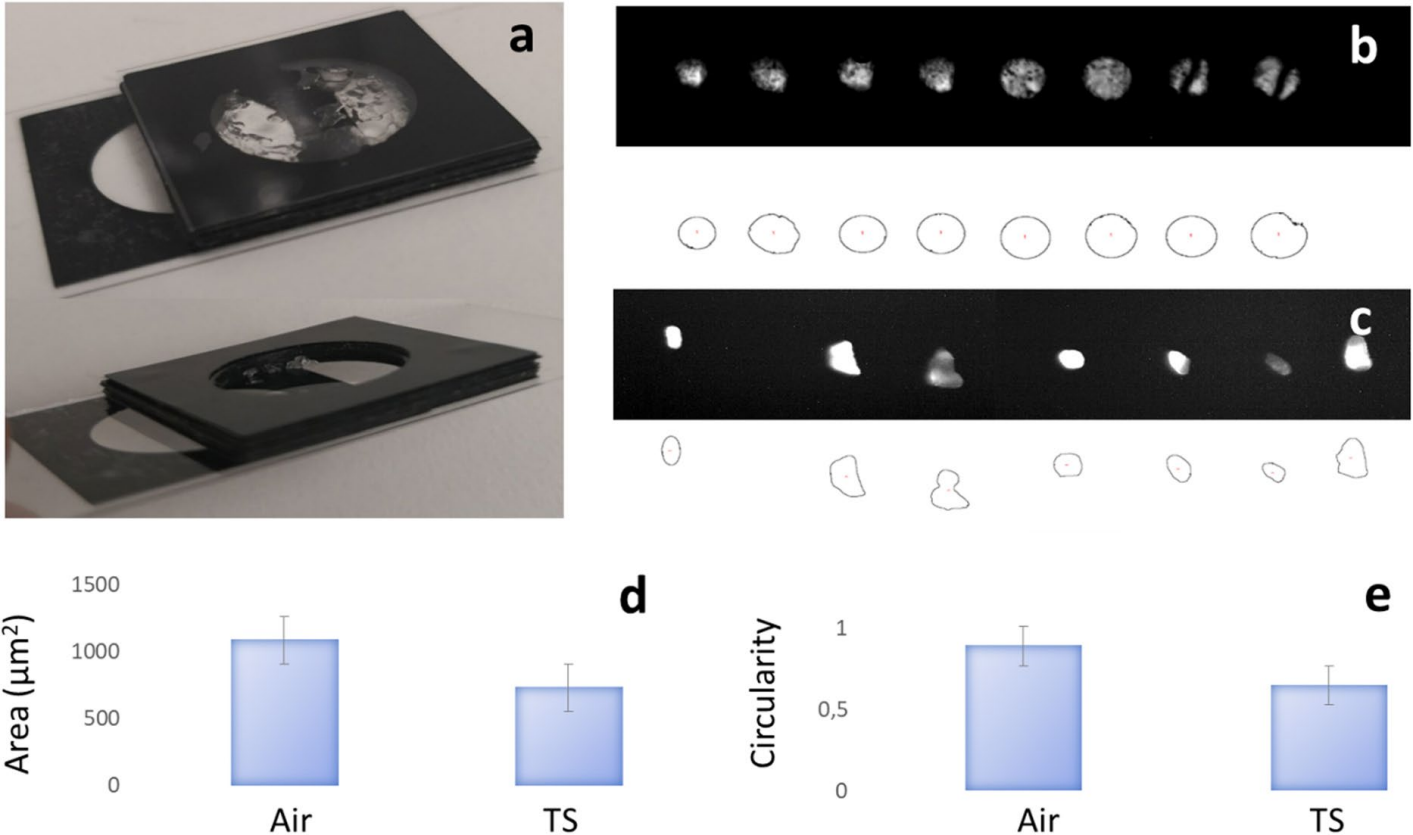
Effect of substrate heterogeneity on the precision of dissection. (**a**) we designed chambers to characterise optical aberrations induced by growth substrates. The chambers are made of cover glass and coverslips separated by vinyl sheets, and they hold a layer of transparent soil and agar of constant thickness. The chambers are then inserted in the path of the laser beam. (**b**)-(**d**) Images of the holes cut into vinyl sheets (top) before thresholding and edge detection using ImageJ (bottom). Images show the substrates have various effects on the dissection. In-air dissections varied in area but kept a circular shape (**b**). When the light passed through a layer of agar, the area cut had altered shape and area (**c**). When the light passed through a layer of transparent soil, the cut also had varied area and shape (**d**). (**e**) The area of the hole cut into the vinyl through agar and TS is significantly reduced compared to the cut obtained through air. The circularity (**f**) of the hole cut into the vinyl was slightly reduced by agar but significantly reduced by transparent soil (TS). Error bars indicate standard errors

Laser cutting was subsequently performed through controlled layers of growth substrates at 0.30 mW to 0.78 mW average power output (Fig. 4a) to study the sensitivity of the dissection to heterogeneity in the medium surrounding plant roots. All experiments carried out in air induced a hole in the vinyl when placed at the focal point of the laser beam (Fig. 4b). The hole created appeared circular and with limited variation in shape. When laser cutting occurred through the agar and transparent soil layers, the holes created in the vinyl changed shape and size (Fig. 4c-d).

Quantitative analysis of the shape of the cut revealed both agar and transparent soil affected the area and circularity of the cut. The area of the hole observed in the vinyl after the beam passed through air (Fig. 4e) was (1.1 ± 0.3) × 10^−3^ mm^2^. The area of the hole observed in the vinyl due to the laser beam passing through agar and transparent soil was (0.7 ± 0.3) × 10^−3^ mm^2^ and (0.7 ± 0.3) × 10^−3^ mm^2^, respectively (Fig. 4e). The area of the cuts achieved in air alone was larger than those achieved through agar and transparent soil but not significantly (p = 0.018 for agar and p = 0.065 for transparent soil, n = 10). Effects were also observed on the circularity of the beam profile (Fig. 4f). The circularity of the hole observed in the vinyl due to the laser beam passing through air was 0.89 ± 0.03, whereas the circularity of the hole observed in the vinyl due to the laser beam passing through agar and transparent soil was 0.79 ± 0.10 and 0.65 ± 0.13 respectively. The circularity of the cuts achieved in air was significantly larger than those achieved through transparent soil (p < 0.001, n = 10), with the differences in the circularity of the cut through agar not significantly different from those observed in samples cut through air (p = 0.017, n = 10). We studied the variability of the position of the centre of mass of the hole to characterise the loss of precision induced by substrate heterogeneity. The variance (eq. 1) measured in transparent soil (65 μm^2^) indicated spatial variations of the cut were small in comparison to the diameter of the cut and therefore, loss of precision was limited.

### Effect of laser output power on the depth of dissection

**Fig. 5:**
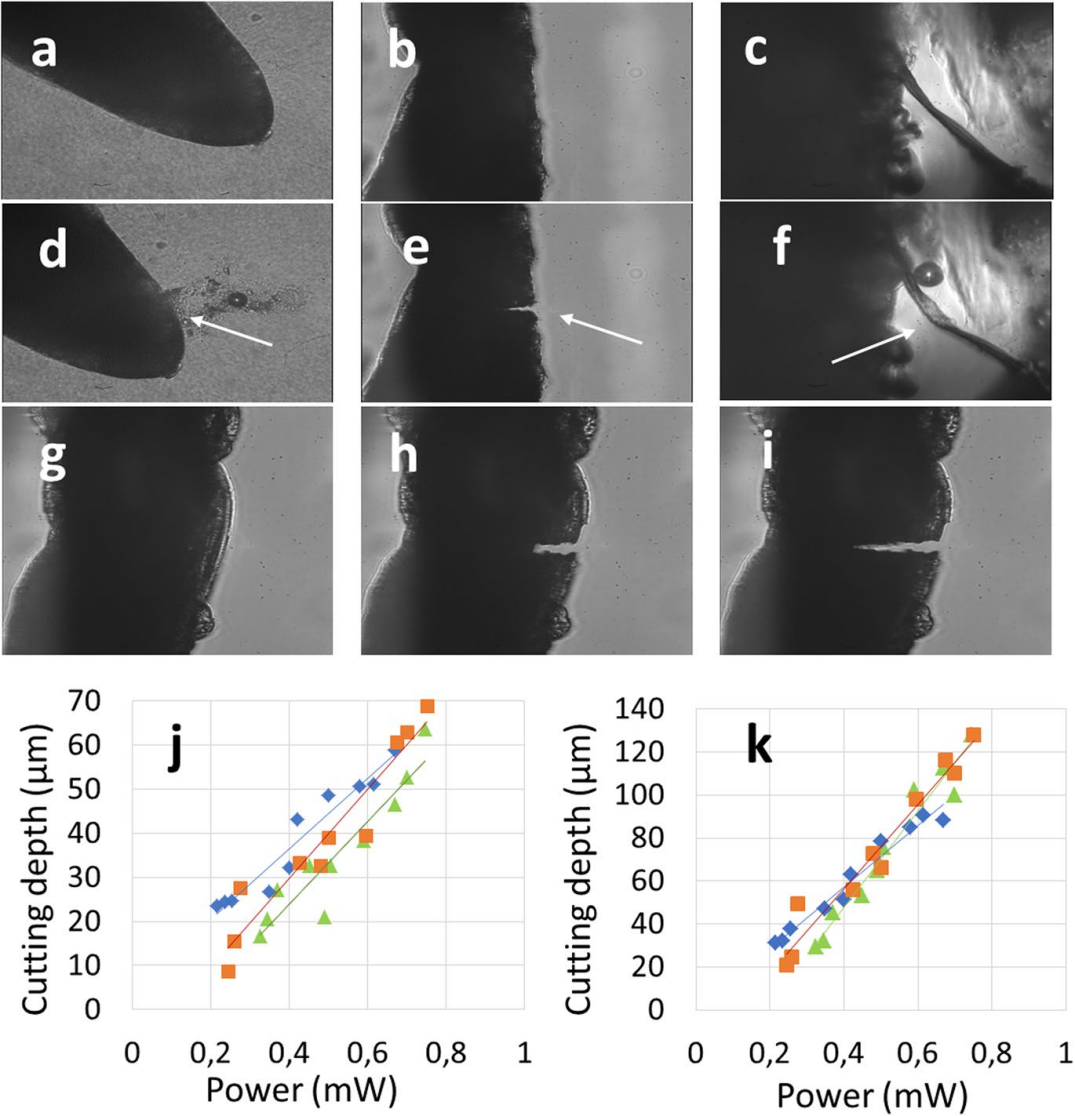
Cutting depth as a function of power output of the laser beam, growth medium and focusing lens. Before the cut, the roots observed in gel (**a**), air (**b**) and transparent soil (**c**) were free of wounds. Application of the laser beam to a root grown in gel induced visible ejecta (**d**). Cuts obtained in air remained opened, probably due to drying (**e**). In transparent soil, removal of tissue material was observed, but the cutting depth could not be measured (**f**). In air the advance of the laser beam into the tissue led to visible and measurable dissection depth (**g-i**). The cutting depth varied between root samples (**j-k**), but there was a limited effect of the focusing lens on the cutting depth of the root tissue

We have tested three different growth environments for the study of the dissection of lettuce roots, namely agar, air and transparent soil (Fig. 5). Dissection in air gave the clearest and most easily quantifiable results and as such could be used as a benchmark for cutting in the other environments. Dissection in agar or transparent soil could be observed, but the depth of the cut wasn’t always easily extracted from optical imaging alone. In agar, for example, ejecta remained in the vicinity of the cut because of reduced mobility of particulates in gel (Fig. 5d). In transparent soil, debris derived from the cut dispersed and were not visible, but measuring the cut was challenging due to swelling and closure of the area dissected by the laser (Fig. 5f). Therefore, the cutting depth was only quantified in air.

The depth of the cut obtained through air was measured following a horizontal scan of the stage inducing a transversal cut at the root apex (Fig. 5g-i). The depth of the cut obtained at a constant speed (74 ± 7) μm s^−1^ was influenced by the power output of the laser beam but not by the characteristics of the focusing lens. In these experiments, the depth of the cut was proportional to the power output of the laser beam, and continuous progression of the beam into the tissue led to a deeper cut (Fig. 5g-i). The depth of the cut increased linearly with the average power output of the laser beam, but tissue dissection was not measurably influenced by the type of focusing lens. Three aspherical lenses were used and showed the cut followed similar relationships between power output of the laser beam and cutting depth (Fig. 5j-k).

### Effect of targeted low light doses on root elongation rate

**Fig. 6:**
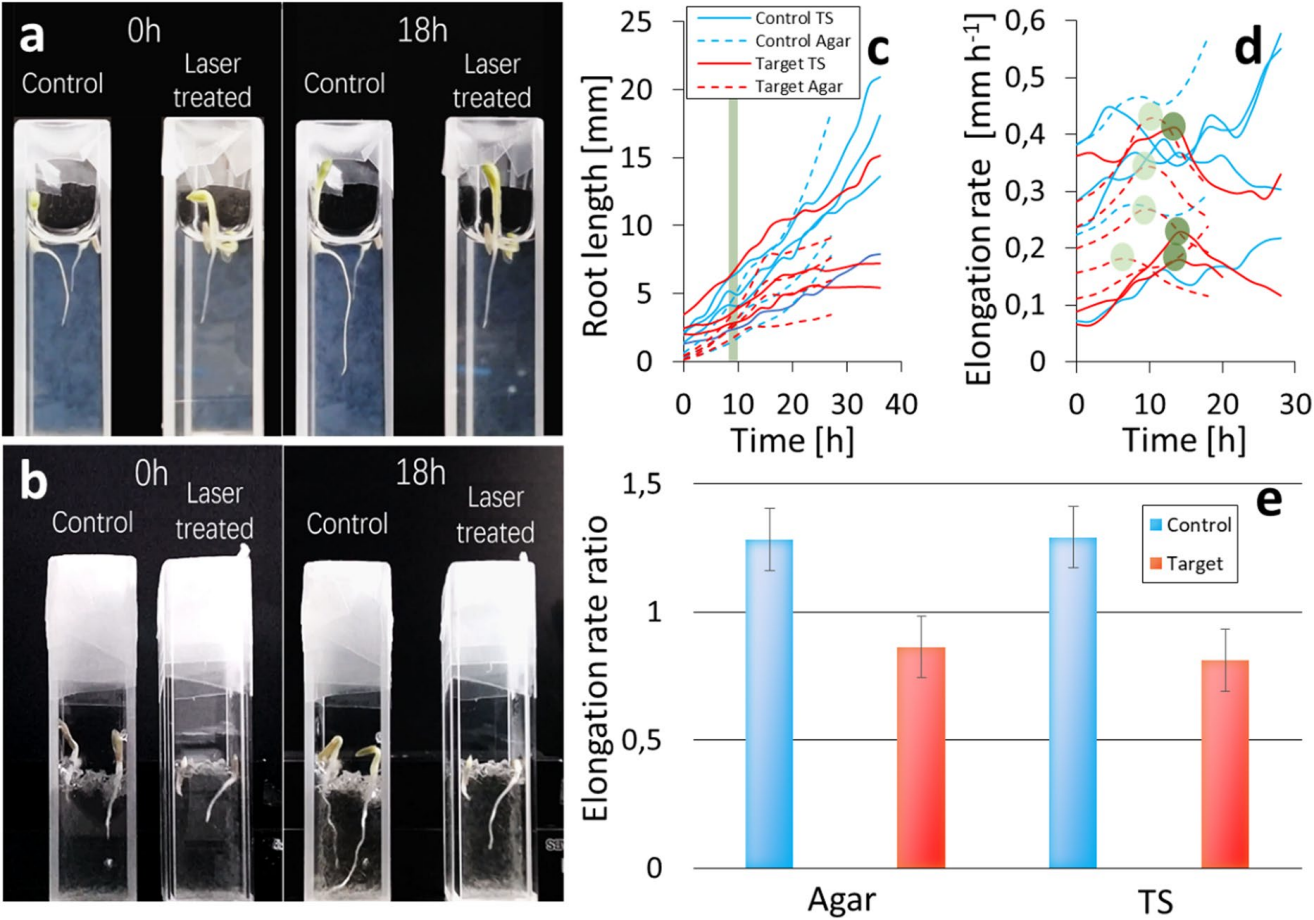
Lower power output from the laser beam reduced the damage to the root, and could be used to modulate the root elongation rate. (**a**) Time-lapse imaging illustrates the effect of laser treatment on the elongation of lettuce roots in agar. The picture on the left shows roots before laser treatment. The picture on the right shows the same roots 18 h following laser treatment. (**b**) Time-lapse imaging illustrates the effect of laser treatment on the elongation of lettuce roots in transparent soil. The picture on the left shows roots before laser treatment. The picture on the right shows laser-treated roots 18 h following laser treatment. (**c**) Root growth curves were observed in gel and transparent soil (n = 4). The green line indicates the time when the laser pulses are applied. Calculation of the root elongation rate (**d**) illustrates the effect of laser treatment on root growth up to 5 h following laser treatment. Dark green discs indicate the location of the inflection point of the response to laser treatment in transparent soil, and light green discs indicate the location of the inflection point of the response to laser treatment in in agar. (**e**) Bar plots of the elongation rate ratio show the growth of laser-treated roots was reduced by approximately 4% in agar and 8% in transparent soil (red) from their elongation rate before exposure. By contrast, the elongation rate of control plants increased by 24% in agar and 38% in transparent soil (blue). Error bars indicate Standard Error

All roots thrived in both agar and transparent soil (Fig. 6a-b). The growth rate was not significantly different between the two treatments (p = 0.66) with rates of (0.25 ± 0.13) mm h^−1^ for roots grown in transparent soil and (0.26 ± 0.10) mm h^−1^ for roots grown in agar.

The elongation rate of roots was affected by the exposure to the laser beam. When roots were not exposed to the laser beam, the root elongation rate increased during the experiment as is consistently observed during organogenesis (blue curves, Fig. 6c) and reached an elongation rate of up to 0.58 mm h^−1^. The average elongation rate observed in roots not exposed to the laser beam was (0.34 ± 0.12) mm h^−1^, and (0.33 ± 0.14) mm h^−1^ for roots grown in transparent soil and agar, respectively (Fig. 6d).

When roots were exposed to a 1 mW laser beam at 150 μm from the focal point, a reduction of the root elongation rate was observed. When roots exposed to the laser grew in agar, the effect of exposure was instantaneous. The reduction in the elongation rate was observed at (9.4 ± 1.6) hours following the start of the experiment and corresponded to the time of the application of the laser. When roots exposed to the laser grew in transparent soil, the reduction in the elongation rate was significantly higher [(14.3 ± 0.6) h, p = 0.008]. The elongation rate of the root was reduced by 8% [(0.23 ± 0.09) mm h^−1^, red solid curves] in transparent soil and by 4% [(0.25 ± 0.10) mm h^−1^, red dotted curves] in agar. By comparison, when roots were not exposed to laser light, the root elongation rate was significantly increased (p < 0.001), with 36% in transparent soil and 27% in agar was observed (Fig. 6e).

## Discussion

### Requirements for laser manipulation of tissues through granular media

Manipulation of biological tissues can be achieved from different types of laser light sources, but achieving desired effects requires good control of physical interactions between the laser light and the sample. In general, pulsed lasers are preferred to continuous-wave lasers in dissection experiments because the reduced interaction times prevent heat dissipation and subsequent tissue damage beyond the target point (Galindo-Castañeda et al. 2018). Amongst pulsed lasers, mode-locked lasers can deliver pulses of ultra-short duration and are often preferred for athermal dissection of tissues (Tirlapur and König 2002). But a mode-locked laser is expensive and as such restricted to specialised laboratories and facilities. In this study, we have tested the application of a Q-switched pulsed laser for the dissection of root tissues, which significantly improve residual damages with comparison to continuous-wave lasers (El-Sherif and King 2003). The pulses achieved with Q-switched laser (microseconds to nanoseconds) are longer in the temporal domains than those reached with mode-locked laser (picoseconds to femtoseconds), but the Q-switched laser only cost around 10% of what would be needed for a mode-locked laser, and we demonstrate manipulations that are robust to focusing lens and substrate heterogeneity.

The path of light is affected at solid-liquid interfaces due to reflection, refraction and scattering (Andrews and Phillips 1998), which can be problematic for laser dissection of roots in situ. In this study, we showed precise root dissection could be achieved, even within the inner structure of the pore space, up to 3 mm inside the transparent soil. Heterogeneities cause changes in the cross-section of the beam but limited spatial variability in the location of the focal point occurs (variance of 65 μm^2^).

No further adjustment of the beam focus (i.e., along the beam axis) was needed because a sufficiently large Rayleigh range was achieved by the instrument through the use of low numerical aperture lenses. Dissection could be achieved with focusing lenses of varying aperture and focal length, which demonstrates the system can be used in a broad range of microscope configurations or applications.

The study also revealed the nature of the constraints to performing laser dissection in situ. Limitations arise first due to the loss of laser power with soil depth. The transmitted power through 1.6 mm of plant growth substrate was reduced by approximately 25% (from 0.41 mW to 0.30 mW). The distortion of the circularity of the beam may be problematic in the deeper soil layers. However, further characterisations of the change in the distribution of the power density within the beam are needed to fully understand limitations to optical cutting at depth. The presence of ejecta (debris produced from the root tissue) also limited subsequent use of live imaging and could obscure the optical path (Shaw 2006). This problem was particularly important when roots were dissected in gel, but less pronounced when roots were dissected in transparent soil (Fig. 5).

### Using laser light for live manipulation of growing tissues

An interesting application of laser manipulation of growing roots is to enable remote control of root elongation without application of chemical substances to the soil such as poly-ethylene glycol or plant growth hormones (Fendrych et al. 2018; Rowe et al. 2016) which are indiscriminate.

To assess whether laser beams can be used to control root elongation in situ, we examined root morphology and root growth following exposure to low light doses. Laser exposure of the apical meristem using low output power (~1 mW at 150 μm distance from the focal point) reduced root elongation rate, and because gel scatters light more than transparent soil (Fig. 4), reductions in root elongation rates observed in transparent soil were larger (8%) than those observed in agar (4%). Also, because the scattering of the gel enlarges the cross-section of the beam (Fig. 4), it was harder to target a small region of the root tip, and broader exposure of the root apical meristem led to immediate arrest of the root elongation. In transparent soil, however, better targeting of the root tip led to effects being observed 4 h after exposure to light, a delay which corresponds well to the time required for meristematic cells to enter the elongation state (Sharp et al. 1988). We did not observe any lesion or tissue damage to root tissues during those experiments. Furthermore, the low output power used to cut or slow down the root and the small volume where this power is reached (beam waist of less than 2 μm and Rayleigh range of less than 20 μm) leave unaffected the vast majority of the soil volume. We therefore conclude that root elongation rate can be controlled in situ using laser beams at low power outputs when focused accurately to the root tip without affecting microbes too much.

Results also suggest larger power outputs can be used to induce lesions and ablate root tissues, without affecting the soil particles. Although the soil scattered the light and modified the circularity of the laser beam, the distortions were minor. The dissections achieved were instantaneous and precise as is typically observed when lasers are used, for example, in mass spectrometry studies (Debeljak et al. 2013). Coupling large scale dissection to live imaging was more challenging because of cutting debris reducing image contrast. Debris is usually removed by gravitational forces, convection/flow, pressurised air or diffusion and dispersion when in the form of liquid, solid particles or vapour (Bernal et al. 2020; Nahen and Vogel 2002; Vogel et al. 2002; Vogel and Venugopalan 2003). When dissection occurs in soil the debris are trapped within soil pores. Their removal requires circulation of liquids to wash the soil continuously during experiments (Banzhaf and Hebig 2016), a process well tested in the transparent soil system when introducing various dyes and refractive index matching liquids (Downie et al. 2012). However, this may not be achievable when using finer soil particles or in gels. The utilisation of more advanced optical manipulation techniques could further expand the use of lasers for in situ manipulations of growing roots. Adaptive optics enables focusing beams more deeply within tissues and other scattering media (Yoon et al. 2018) and would improve beam quality at depth in transparent soil for laser dissection. Wavefront control, in particular, could be used to pre-correct for aberrations caused by soil heterogeneity (Kanngiesser and Roth 2020) by maintaining the circularity and trapping gradient at a clear beam waist deep within the soil. Computer-controlled Spatial Light Modulators (SLM) for example, have been used to increase the focal depth and to minimise aberrations observed in a range of materials (Čižmár et al. 2010), and the technique has been well tested in microsurgery (Jayasinghe et al. 2011). Micromirror devices have also been used for deep tissue imaging (Wang et al. 2015).

The management of residues produced by laser dissection could also be improved. Fine tuning of the cutting speed and temperature control of the tissue are commonly used to manage laser dissection ejecta (Bernal et al. 2020). Liquid or gas jets can also be used to control the flow of ejecta (Debonnel et al. 2003), but this approach is difficult to apply when dissection occurs in soil. It is possible, however, that development in microfluidics will unlock current difficulties of managing debris generated in soil. Current chips now enable to achieve fine control of where roots grow in cultivation chambers (Horade et al. 2012), and also to facilitate separation and removal of materials from samples for analysis (Tetala et al. 2009).

### Opportunities for optical manipulation techniques in plant and soil studies

To date, the use of lasers in plant and soil sciences has largely focused on imaging studies. Because laser light sources are collimated, they can selectively illuminate planes or points within a living specimen and enable fast reconstructions of root and soil structures in situ with minimum photodamage. This is the case of laser scanners that reconstruct 3D root architectures (Fang et al. 2009), or light-sheet (Yang et al. 2013) and confocal laser scanning microscopes (Bengough et al. 2010), which monitor root growth in response to the physical environment. A number of imaging techniques also exploit the coherence and high peak power of lasers to extract biological or biochemical signals deeper into tissue, for example using multiphoton fluorescence (Bureau et al. 2018), Raman microscopy (Soukup et al. 2017), or to obtain indicators of biological activity without the use of dyes (Ribeiro et al. 2014).

Fundamental research has made extensive use of laser dissection to address questions related to cell biology and organogenesis. Because focused laser beams can remove regions of tissues of few microns in size, the technique can be used to test the function of specific cell types in meristems (Reinhardt et al. 2003; Xu et al. 2006). The development of Laser Ablation Tomography (Strock et al. 2019) however has recently opened avenues of research on the application of lasers to study root interactions with the soil environment. The technique does not require sophisticated optical instruments, it uses optics with low numerical aperture and requires only a short operation time, it allows root anatomical studies of large samples at higher-throughput than conventional sectioning using a microtome (Vitha et al. 2000). It has been used, for example, to study genotypic responses to soil mechanical resistance (Chimungu et al. 2015), to study root anatomical adaptation to drought (Hazman and Brown 2018) or to study how pathogens colonise the inner structure of roots (Strock et al. 2019).

In situ laser manipulation of root tissues further expands our abilities to address scientific questions related to the biophysics of root-soil interactions. For example, dissection of plant tissues is used to measure the mechanical stresses generated during growth (Dumais and Steele 2000). The deformation of a lesion following dissection is used to determine whether tissues are in tension or compression, and to determine the magnitude of the stress in the tissue. Applying such methods to roots grown in soil would reveal how mechanical stress accumulates in plant roots when growing against impeding soils impedance and how roots find paths of least resistance (Bengough and Mullins 1990; Martins et al. 2020). With further development of techniques to manage debris created by dissection, it would also be possible to perform Laser Ablation Tomography in situ, so that mechanical stress in the root can also be associated to anatomical features.

Rhizosphere biology is another important field of research which could benefit from in situ laser micromanipulation. Microbial colonisation of roots, in particular, is challenging to understand because interactions with roots are rare, stochastic, or affected by numerous anatomical, biochemical and physiological factors (Palmer et al. 2007). A direct application of the work presented here would be to study how root elongation rate affects the level of colonisation of the root (Dupuy and Silk 2016). Targeted exposure to low light doses (Fig. 6) could be used to control the elongation while simultaneously recording the level of bacterial colonisation on the rhizoplane. In situ laser dissection would also enable studies of chemotactic response and subsequent internalisation of pathogens following the creation of lesions (Faulkner and Robatzek 2012), or to study the role of specific cell types on the attachment of bacteria on root surfaces (Humphris et al. 2005). Furthermore, the development of multiphoton excitation indicates the possibility to directly ablate on specific cells inside the plant without affecting the surface (Galbraith and Terasaki 2003).

## Conclusions

We demonstrate the successful application of dissection and manipulation of roots within the inner structure of artificial soils. This was achieved using a simple optical set up which could easily be introduced to a variety of microscopes. The technique was shown to be robust to aberrations induced by transparent soil such that aberration correction was not needed to improve the positioning of the focus of the beam. The application of laser dissection techniques to soil was easily controlled through parameters such as output power of the laser beam and distance from focal point. The technique opens new avenues of research in rhizosphere biology, with applications such as trapping (Paterson et al. 2001), force measurements (Dholakia et al. 2002) and controlled displacement of cells (Zhang and Liu 2008) also within reach.

## Data Availability

Not applicable.

## References

[CR1] Abràmoff MD, Magalhães PJ, Ram SJ (2004). Image processing with ImageJ. Biophoton Int.

[CR2] Adu MO, Chatot A, Wiesel L, Bennett MJ, Broadley MR, White PJ, Dupuy LX (2014). A scanner system for high-resolution quantification of variation in root growth dynamics of Brassica rapa genotypes. J Exp Bot.

[CR3] Andrews L, Phillips R (1998) Laser beam propagation through random media

[CR4] Banzhaf S, Hebig KH (2016). Use of column experiments to investigate the fate of organic micropollutants–a review. Hydrol Earth Syst Sci.

[CR5] Bengough AG, Mullins CE (1990). Mechanical impedance to root growth: a review of experimental techniques and root growth responses. J Soil Sci.

[CR6] Bengough AG, Hans J, Bransby MF, Valentine TA (2010). PIV as a method for quantifying root cell growth and particle displacement in confocal images. Microsc Res Tech.

[CR7] Bengough AG, McKenzie B, Hallett P, Valentine T (2011). Root elongation, water stress, and mechanical impedance: a review of limiting stresses and beneficial root tip traits. J Exp Bot.

[CR8] Bernal LMB, Canbaz F, Droneau A, Friederich NF, Cattin PC, Zam A (2020). Optimizing deep bone ablation by means of a microsecond Er: YAG laser and a novel water microjet irrigation system. Biomedical Optics Express.

[CR9] Boyd RW, Lukishova SG, Shen YR (2008) Self-focusing: Past and Present: Fundamentals and Prospects, vol 114. Springer Science & Business Media, Berlin

[CR10] Bureau C, Lanau N, Ingouff M, Hassan B, Meunier A-C, Divol F, Sevilla R, Mieulet D, Dievart A, Périn C (2018). A protocol combining multiphoton microscopy and propidium iodide for deep 3D root meristem imaging in rice: application for the screening and identification of tissue-specific enhancer trap lines. Plant Methods.

[CR11] Chimungu JG, Loades KW, Lynch JP (2015). Root anatomical phenes predict root penetration ability and biomechanical properties in maize (Zea mays). J Exp Bot.

[CR12] Čižmár T, Mazilu M, Dholakia K (2010). In situ wavefront correction and its application to micromanipulation. Nat Photonics.

[CR13] De Vries AH, Krenn BE, van Driel R, Kanger JS (2005). Micro magnetic tweezers for nanomanipulation inside live cells. Biophys J.

[CR14] Debeljak M, van Elteren JT, Vogel-Mikuš K (2013). Development of a 2D laser ablation inductively coupled plasma mass spectrometry mapping procedure for mercury in maize (Zea mays L.) root cross-sections. Anal Chim Acta.

[CR15] Debonnel C, Yu S, Peterson P (2003). X-ray ablation and debris venting for the HIF point design. Fusion Sci Technol.

[CR16] Dholakia K, MacDonald M, Spalding G (2002). Optical tweezers: the next generation. Physics World.

[CR17] Downie H, Holden N, Otten W, Spiers AJ, Valentine TA, Dupuy LX (2012). Transparent soil for imaging the rhizosphere. PLoS One.

[CR18] Dumais J, Steele CR (2000). New evidence for the role of mechanical forces in the shoot apical meristem. J Plant Growth Regul.

[CR19] Dupuy LX, Silk WK (2016) Mechanisms of early microbial establishment on growing root surfaces. Vadose Zone J 15(2):vzj2015. 2006.0094

[CR20] El-Sherif A, King T (2003). Soft and hard tissue ablation with short-pulse high peak power and continuous thulium-silica fibre lasers. Lasers Med Sci.

[CR21] Fang S, Yan X, Liao H (2009). 3D reconstruction and dynamic modeling of root architecture in situ and its application to crop phosphorus research. Plant J.

[CR22] Faulkner C, Robatzek S (2012). Plants and pathogens: putting infection strategies and defence mechanisms on the map. Curr Opin Plant Biol.

[CR23] Fendrych M, Akhmanova M, Merrin J, Glanc M, Hagihara S, Takahashi K, Uchida N, Torii KU, Friml J (2018). Rapid and reversible root growth inhibition by TIR1 auxin signalling. Nature Plants.

[CR24] Gadalla A, Dehoux T, Audoin B (2014). Transverse mechanical properties of cell walls of single living plant cells probed by laser-generated acoustic waves. Planta.

[CR25] Galbraith JA, Terasaki M (2003). Controlled damage in thick specimens by multiphoton excitation. Mol Biol Cell.

[CR26] Galindo-Castañeda T, Brown KM, Lynch JP (2018). Reduced root cortical burden improves growth and grain yield under low phosphorus availability in maize. Plant Cell Environ.

[CR27] Gomez EF, Berggren M, Simon DT (2017). Surface acoustic waves to drive plant transpiration. Sci Rep.

[CR28] Gregory PJ, Hutchison D, Read DB, Jenneson PM, Gilboy WB, Morton EJ (2003) Non-invasive imaging of roots with high resolution X-ray micro-tomography. In: roots: the dynamic interface between plants and the earth. Springer, pp 351-359

[CR29] Hazman M, Brown KM (2018). Progressive drought alters architectural and anatomical traits of rice roots. Rice.

[CR30] Hodge A, Berta G, Doussan C, Merchan F, Crespi M (2009). Plant root growth, architecture and function. Plant Soil.

[CR31] Horade M, Mizuta Y, Kaji N, Higashiyama T, Arata H (2012) Plant-on-a-chip microfluidic-system for quantitative analysis of pollen tube guidance by signaling molecule: towards cell-to-cell communication study. In: Proc microTAS, pp. 1027–1029

[CR32] Humphris SN, Bengough AG, Griffiths BS, Kilham K, Rodger S, Stubbs V, Valentine TA, Young IM (2005). Root cap influences root colonisation by Pseudomonas fluorescens SBW25 on maize. FEMS Microbiol Ecol.

[CR33] Jayasinghe AK, Rohner J, Hutson MS (2011). Holographic UV laser microsurgery. Biomed Optics Expr.

[CR34] Kanngiesser J, Roth B (2020). Wavefront shaping concepts for application in optical coherence tomography—a review. Sensors.

[CR35] Li Y, Xin H, Liu X, Li B (2015). Non-contact intracellular binding of chloroplasts in vivo. Sci Rep.

[CR36] Martins AD, O'Callaghan F, Bengough AG, Loades KW, Pasqual M, Kolb E, Dupuy LX (2020). The helical motions of roots are linked to avoidance of particle forces in soil. New Phytol.

[CR37] Nahen K, Vogel A (2002). Plume dynamics and shielding by the ablation plume during Er: YAG laser ablation. J Biomed Opt.

[CR38] Nakazono M, Qiu F, Borsuk LA, Schnable PS (2003). Laser-capture microdissection, a tool for the global analysis of gene expression in specific plant cell types: identification of genes expressed differentially in epidermal cells or vascular tissues of maize. Plant Cell.

[CR39] O’Callaghan FE, Braga RA, Neilson R, MacFarlane SA, Dupuy LX (2018). New live screening of plant-nematode interactions in the rhizosphere. Sci Rep.

[CR40] Palmer J, Flint S, Brooks J (2007). Bacterial cell attachment, the beginning of a biofilm. J Ind Microbiol Biotechnol.

[CR41] Paterson L, MacDonald MP, Arlt J, Sibbett W, Bryant P, Dholakia K (2001). Controlled rotation of optically trapped microscopic particles. Science.

[CR42] Reinhardt D, Frenz M, Mandel T, Kuhlemeier C (2003) Microsurgical and laser ablation analysis of interactions between the zones and layers of the tomato shoot apical meristem 10.1242/dev.00596 12874128

[CR43] Ribeiro K, Barreto B, Pasqual M, White P, Braga R, Dupuy LX (2014). Continuous, high-resolution biospeckle imaging reveals a discrete zone of activity at the root apex that responds to contact with obstacles. Ann Bot.

[CR44] Romano I, Ventorino V, Pepe O (2020). Effectiveness of plant beneficial microbes: overview of the methodological approaches for the assessment of root colonization and persistence. Front Plant Sci.

[CR45] Rowe JH, Topping JF, Liu J, Lindsey K (2016). Abscisic acid regulates root growth under osmotic stress conditions via an interacting hormonal network with cytokinin, ethylene and auxin. New Phytol.

[CR46] Rudolph-Mohr N, Vontobel P, Oswald SE (2014). A multi-imaging approach to study the root–soil interface. Ann Bot.

[CR47] Schou J, Heisel T, Nordskov A, Christensen S, Jensen PS, Thestrup B, Toftmann B (2002) Quantitative laser cutting of plants. In: High-Power Laser Ablation IV. International Society for Optics and Photonics, pp 734–742

[CR48] Sharma K, Palatinszky M, Nikolov G, Berry D, Shank EA (2020). Transparent soil microcosms for live-cell imaging and non-destructive stable isotope probing of soil microorganisms. Elife.

[CR49] Sharp RE, Silk WK, Hsiao TC (1988). Growth of the maize primary root at low water potentials: I. spatial distribution of expansive growth. Plant Physiol.

[CR50] Shaw SL (2006). Imaging the live plant cell. Plant J.

[CR51] Soukup M, Martinka M, Bosnić D, Čaplovičová M, Elbaum R, Lux A (2017). Formation of silica aggregates in sorghum root endodermis is predetermined by cell wall architecture and development. Ann Bot.

[CR52] Strock CF, Schneider HM, Galindo-Castañeda T, Hall BT, Van Gansbeke B, Mather DE, Roth MG, Chilvers MI, Guo X, Brown K (2019). Laser ablation tomography for visualization of root colonization by edaphic organisms. J Exp Bot.

[CR53] Tam JM, Castro CE, Heath RJ, Cardenas ML, Xavier RJ, Lang MJ, Vyas JM (2010). Control and manipulation of pathogens with an optical trap for live cell imaging of intercellular interactions. PLoS One.

[CR54] Tetala KK, Swarts JW, Chen B, Janssen AE, van Beek TA (2009). A three-phase microfluidic chip for rapid sample clean-up of alkaloids from plant extracts. Lab Chip.

[CR55] Tirlapur UK, König K (2002). Femtosecond near-infrared laser pulses as a versatile non-invasive tool for intra-tissue nanoprocessing in plants without compromising viability. Plant J.

[CR56] Tracy SR, Black CR, Roberts JA, Sturrock C, Mairhofer S, Craigon J, Mooney SJ (2012). Quantifying the impact of soil compaction on root system architecture in tomato (Solanum lycopersicum) by X-ray micro-computed tomography. Ann Bot.

[CR57] Unger PW, Kaspar TC (1994). Soil compaction and root growth: a review. Agron J.

[CR58] van Dusschoten D, Metzner R, Kochs J, Postma JA, Pflugfelder D, Bühler J, Schurr U, Jahnke S (2016). Quantitative 3D analysis of plant roots growing in soil using magnetic resonance imaging. Plant Physiol.

[CR59] Vitha S, Baluška F, Jasik J, Volkmann D, Barlow PW (2000) Steedman’s wax for F-actin visualization. In: actin: a dynamic framework for multiple plant cell functions. Springer, pp 619-636

[CR60] Vogel A, Venugopalan V (2003). Mechanisms of pulsed laser ablation of biological tissues. Chem Rev.

[CR61] Vogel A, Schmidt P, Flucke B (2002) Minimization of thermomechanical side effects and increase of ablation efficiency in IR ablation by use of multiply Q-switched laser pulses. In: Laser Tissue Interaction XIII: Photochemical, Photothermal, and Photomechanical. International Society for Optics and Photonics, pp 105–111

[CR62] Wang D, Zhou EH, Brake J, Ruan H, Jang M, Yang C (2015). Focusing through dynamic tissue with millisecond digital optical phase conjugation. Optica.

[CR63] Xu J, Hofhuis H, Heidstra R, Sauer M, Friml J, Scheres B (2006). A molecular framework for plant regeneration. Science.

[CR64] Yang Z, Downie H, Rozbicki E, Dupuy LX, MacDonald MP (2013). Light sheet tomography (LST) for in situ imaging of plant roots. Opt Express.

[CR65] Yoon T, Kim C-S, Kim K, Choi J-r (2018). Emerging applications of digital micromirror devices in biophotonic fields. Opt Laser Technol.

[CR66] Zhang H, Liu K-K (2008). Optical tweezers for single cells. J R Soc Interface.

